# Characterizing Weibo Social Media Posts From Wuhan, China During the Early Stages of the COVID-19 Pandemic: Qualitative Content Analysis

**DOI:** 10.2196/24125

**Published:** 2020-12-07

**Authors:** Qing Xu, Ziyi Shen, Neal Shah, Raphael Cuomo, Mingxiang Cai, Matthew Brown, Jiawei Li, Tim Mackey

**Affiliations:** 1 Department of Healthcare Research and Policy University of California, San Diego - Extension La Jolla, CA United States; 2 Global Health Policy and Data Institute San Diego, CA United States; 3 S-3 Research LLC San Diego, CA United States; 4 Masters Program in Computer Science Jacobs School of Engineering University of California, San Diego La Jolla, CA United States; 5 Department of Anesthesiology School of Medicine University of California, San Diego La Jolla, CA United States; 6 US Embassy National Cancer Institute National Institutes of Health Beijing China; 7 Department of Anesthesiology and Division of Infectious Diseases and Global Public Health School of Medicine University of California, San Diego La Jolla, CA United States

**Keywords:** COVID-19, infodemiology, infoveillance, infodemic, Weibo, social media, content analysis, China, data mining, knowledge, attitude, behavior

## Abstract

**Background:**

The COVID-19 pandemic has reached 40 million confirmed cases worldwide. Given its rapid progression, it is important to examine its origins to better understand how people’s knowledge, attitudes, and reactions have evolved over time. One method is to use data mining of social media conversations related to information exposure and self-reported user experiences.

**Objective:**

This study aims to characterize the knowledge, attitudes, and behaviors of social media users located at the initial epicenter of the outbreak by analyzing data from the Sina Weibo platform in Chinese.

**Methods:**

We used web scraping to collect public Weibo posts from December 31, 2019, to January 20, 2020, from users located in Wuhan City that contained COVID-19–related keywords. We then manually annotated all posts using an inductive content coding approach to identify specific information sources and key themes including news and knowledge about the outbreak, public sentiment, and public reaction to control and response measures.

**Results:**

We identified 10,159 COVID-19 posts from 8703 unique Weibo users. Among our three parent classification areas, 67.22% (n=6829) included news and knowledge posts, 69.72% (n=7083) included public sentiment, and 47.87% (n=4863) included public reaction and self-reported behavior. Many of these themes were expressed concurrently in the same Weibo post. Subtopics for news and knowledge posts followed four distinct timelines and evidenced an escalation of the outbreak’s seriousness as more information became available. Public sentiment primarily focused on expressions of anxiety, though some expressions of anger and even positive sentiment were also detected. Public reaction included both protective and elevated health risk behavior.

**Conclusions:**

Between the announcement of pneumonia and respiratory illness of unknown origin in late December 2019 and the discovery of human-to-human transmission on January 20, 2020, we observed a high volume of public anxiety and confusion about COVID-19, including different reactions to the news by users, negative sentiment after being exposed to information, and public reaction that translated to self-reported behavior. These findings provide early insight into changing knowledge, attitudes, and behaviors about COVID-19, and have the potential to inform future outbreak communication, response, and policy making in China and beyond.

## Introduction

First documented in December 2019, the novel coronavirus is thought to have originated from the city of Wuhan in Hubei Province, China and has quickly emerged as the greatest global public health threat in the last century while also representing a significant test for global preparedness to prevent, diagnose, treat, and contain a highly transmittable disease. At the end of October 2020, this novel coronavirus (COVID-19) pandemic has now impacted 189 countries and geographic territories, with over 40 million confirmed cases and rising, accompanied by over 1 million global deaths [1]. This includes over 91,772 confirmed cases from China [2], with 68,139 cases that originated in Wuhan and 50,340 cases that originated in the initial epicenter of the outbreak in Wuhan City [3,4].

With the COVID-19 outbreak originating and spreading from China, notable given the country’s large population, high density of Hubei Province, and the outbreak coinciding with the Lunar New Year period, China implemented a significant public health response including mandated quarantines, community and social isolation, and the construction of two new hospitals [5]. Despite these aggressive measures, at the onset of the outbreak, little was known about the structure, etiology, transmission dynamics, and appropriate public health measures needed to curtail the spread of COVID-19. Much of the fundamental understanding of COVID-19, including the fact that it was a novel coronavirus, emerged throughout the month of January, as the outbreak rapidly spread throughout mainland China.

With the COVID-19 outbreak now a global pandemic, there is a critical need to better understand the origins of the disease and what lessons can be learned from public reaction due to information exposure, or lack thereof, and people’s subsequent responses to public health measures implemented. One such approach is the use of data in an electronic medium to supplement traditional epidemiological surveillance measures, also knowns as “infoveillance” [6]. Data derived from social media platforms represents one of these infoveillance data layers and can be collected and analyzed for the purposes of gauging the public’s knowledge, attitudes, and behavior in close to real time. This includes past research leveraging social media to better assess public reaction to outbreaks such as H1N1, Zika virus, and Ebola [7-12].

In the context of the early stages of COVID-19, infoveillance approaches examining popular Chinese social media sites are needed, as most global social media platforms (eg, Twitter, Facebook, Instagram, and Reddit) are blocked in China. As a result, Chinese social media activity primarily occurs on two platforms: WeChat and Sina Weibo. WeChat is a popular messaging service in China where users can privately communicate with one another, whereas Sina Weibo (commonly referred to as just “Weibo” by its estimated 480 million active users) more closely resembles a microblogging platform with public posts. In 2018, 57% of Weibo users were reported as male and 43% as female [13]. In terms of age, the largest group of users (40%) are aged 23-30 years, followed by users aged 18-22 years (35%), 31-40 years (14%), and 16-17 years (6%); only 5% of users are older than 41 years [13]. These demographics generally skew similar to other popular microblogging platforms such as Twitter whose features and purpose are directly comparable. Specifically, Weibo users can post and interact publicly with information as it arises, representing an accessible and important infoveillance data source to characterize user experiences and conversations during different stages of COVID-19.

Leveraging this data source, other studies have used Weibo posts to characterize conversations and analyze public sentiment, including evaluating whether aspects of the COVID-19 outbreak can be better modeled or characterized [14-21]. One study assessed user-generated discussions on Weibo and found there was public demand for appropriate health resources and equipment during early outbreak stages [22]. Previous studies by our group have identified prevailing COVID-19 themes discussed among Wuhan Weibo users, including uncertainty about the origins of the outbreak, changing concerns about disease transmission characteristics, and varying reactions to the government’s outbreak response. Other studies have developed models to better gauge public opinion about COVID-19 among Chinese Weibo users and assess whether adequate warning and attention was given to the outbreak by authorities [17]. Finally, a recent study used infoveillance approaches to track how internet searches and Weibo discussions peaked prior to increased daily case incidence data, and another study used Weibo to identify characteristics of suspected COVID-19 cases and their help-seeking behavior [18,23].

Our study seeks to add to this growing body of literature by leveraging Weibo to better understand Chinese user sentiment and behavior associated with the COVID-19 pandemic. Specifically, the objective of this study is to conduct an in-depth qualitative analysis of Chinese language Weibo posts originating from users located in Wuhan during the early periods of the outbreak to characterize types of news and user knowledge that may have coincided with changes in public sentiment and reaction to the outbreak. This study has an interdisciplinary approach, using methods in computer science, public health, and qualitative analysis.

## Methods

### Data Collection

To fully explore early COVID-19 outbreak topics and information shared and disseminated by the government, media, and Chinese users in Wuhan, this study first collected social media posts on Sina Weibo and then conducted in-depth qualitative content analysis of all posts collected. Qualitative analysis was used for the purposes of identifying and characterizing user knowledge, attitudes, and impact on behavior from the initial announcement of the pneumonia of unknown origin until the implementation of quarantine in Wuhan City. The overall aim of this study is to identify and characterize key thematic discussions and public reaction located at ground zero of this global pandemic.

Beginning December 31, 2019, we employed an automated web scraper built in the programming language Python to collect public posts on the Weibo platform in the Chinese language (traditional and simplified Chinese). This programming script was set to collect time and geographically filtered data using preset settings available on the advanced search function of the platform. Filters included COVID-19–related keywords, a geographic limitation to collect posts from users located in Wuhan City, and a time period spanning December 31, 2019, to January 20, 2020. Chinese language COVID-19–related keywords were used as filters for this study and included 新型冠状病毒 (novel coronavirus), 武汉肺炎 (Wuhan pneumonia), 不明原因肺炎 (unknown caused pneumonia), and other words associated with the outbreak during its early stages: 华南海鲜市场 (Wuhan Seafood Wholesale Market), and severe acute respiratory syndrome (SARS). Data collected included textual content of post, user and account information, and date and time of post. Messages collected for the purposes of this study were in the public domain and web scraping was done solely for research purposes. Our study does not disclose any personally identifiable information, as we have removed identifiers from our data set and aggregated results. Hence, no ethics approval was required for this study, as we relied on publicly available data, did not include any identifiable information, did not include any private messages between users, and there was no interaction between Weibo users and researchers.

### Qualitative Content Analysis

Weibo permits users to post up to 2000 characters with or without images, videos, and other multimedia, and users may also repost messages. These characteristics (particularly the high character count compared to other microblogging platforms) permits a users’ post to address multiple topics and have a rich qualitative discussion of issues. To fully capture and accurately classify topics discussed on Weibo related to COVID-19, we manually annotated all posts collected by conducting content analysis of the text in the posts.

The focus of our content analysis was to detect themes associated with knowledge, attitudes, and beliefs of Chinese social media users located in Wuhan in response to exposure to government information sources; news and media outlets; and, specifically, reaction to events that occurred as the outbreak evolved in the local, national, and global context. Specific themes of interest included conversations and user reactions related to public anxiety, confusion, concerns, and behavior adaptation based on COVID-19–related developments.

Content analysis was conducted using an inductive coding approach primarily because there are few existing qualitative analyses of COVID-19 data in the Chinese language [17,24]. Hence, this open coding approach allowed us to create our own coding classifications based directly on Weibo posts observed. This was accomplished by organizing the data set into binned samples stratified over the entire study period, conducting a first round of content coding based on a generalized sample of the binned data set, creating an initial codebook of parent and subclassifications, rereading the samples and applying codes and classifications, and repeating these steps until all data was coded.

First, two coders (first and second authors) independently used a binary coding approach (ie, relevant vs nonrelevant) to filter posts related to COVID-19 and exclude posts not related to the outbreak. We then used thematic content analysis coding methods, with two coders randomly selecting 200 posts that were then independently coded for parent topic classifications to represent baseline thematic areas or interest and for the purposes of collapsing overlapping or infrequent categories. We then combined the independently coded data sets and created a codebook based on these initial classifications. Coders then independently manually annotated all posts collected in this study and categorized new messages into identified parent topics and existing and new subtopics. Through this process, we selected parent and subtopic classifications, and collapsed infrequent categories by combining related topics, removing duplicate topics, and evaluating thematic concurrence.

We first identified posts for general relevance that we binarily coded as either related or not related to COVID-19 topics. After excluding nonrelevant posts and considering the underlining structure of the Weibo posts, we categorized posts into three broad information source classifications: (1) posts that contained only information from government or news and media sources, (2) posts that contained only user-generated comments, and (3) posts that contained both government/news, and user-generated comments.

We then summarized the content of posts for three parent classification areas initially identified in our random sample of time stratified posts: (1) news and knowledge about the outbreak, (2) public sentiment of users to the outbreak (using an initial set of Chinese language polarity lexicons that were iterated upon, including positive, negative, and neutral [25], and chosen on their basis of relevance to our open inductive coding approach and focus on health topics; see Textbox 1), and (3) public reaction to control and response measures. Subclassification of topics within these parent classifications were based on the Health Belief Model, where constructs of perceived susceptibility and severity, and cues for action were explored for user-generated posts [26,27]. Before analyzing the text of each post and to account for a single post covering multiple topics, we separated messages into different topic groupings based on the aforementioned parent classifications. This included separating out content based on the information source.

We also assessed other characteristics of Weibo posts, including temporal variations among topics that occurred over time, and assessed reactions to posts by other users. By analyzing the relative number and content of users’ reaction to posts, we could observe variation in the public’s attitude from the beginning of the COVID-19 outbreak, as well as measure how these reactions changed over time specific to news and events that emerged as the outbreak progressed. For both public sentiment and public reaction classifications, temporal stationarity was assessed using the augmented Dickey-Fuller test. For nonstationary categories, regression models were then built to determine statistical significance of linear and exponential relationships, with linear fit (linear *R*^2^) and exponential fit (Cox and Snell *R*^2^) compared for relationships under the threshold α=.05.

**List of positive, neutral, and negative lexicons.**

**Positive lexicon**

- Perfect (完美)
- Practical (实用)
- Powerful (强大)
- Good (好)
- Clear (清晰)
- Reliable (可靠的)
- Believable (值得信任的)
- Authoritative (权威性的)
- Confident (自信的/有信心的)
- Fast (快的)
- Safe (安全的)

**Neutral lexicon**

- Known (知道/听说)
- Suggested (提出的)
- Investigative (调查的)
- Announced (公布)
- Confirmed (确认的)
- Existing (存在)
- Quiet (安静的)
- Unconcerned (不在乎)

**Negative lexicon**

- Bad (差)
- Poor (烂)
- Awful (坏)
- Disappoint (失望)
- Slow (慢)
- Not work (不行)
- Scared (可怕的)
- Fake (假的)
- Angry (生气的)
- Unclear (模糊的)
- Nervous (紧张的)

## Results

### Data

We collected 10,814 Weibo posts located in Wuhan over the 21-day study period. After coding all of these posts independently, the first and second coders achieved a relatively high intercoder reliability score for results (κ=0.892). Disagreements in coding classification were reviewed by first and second author, and discussed to reach consensus on correct classification. The oldest post collected was dated December 31, 2019, at 12:31 AM (China standard time), and the last post collected was dated January 20, 2020, at 11:00 PM. After manually annotating posts, we filtered out 655 posts (6.06% of the total data set) not related to the COVID-19 outbreak and retrieved 10,159 posts (93.94%) that we identified as directly related to COVID-19 discussions that comprised of 8703 unique Weibo users.

**Figure 1:**
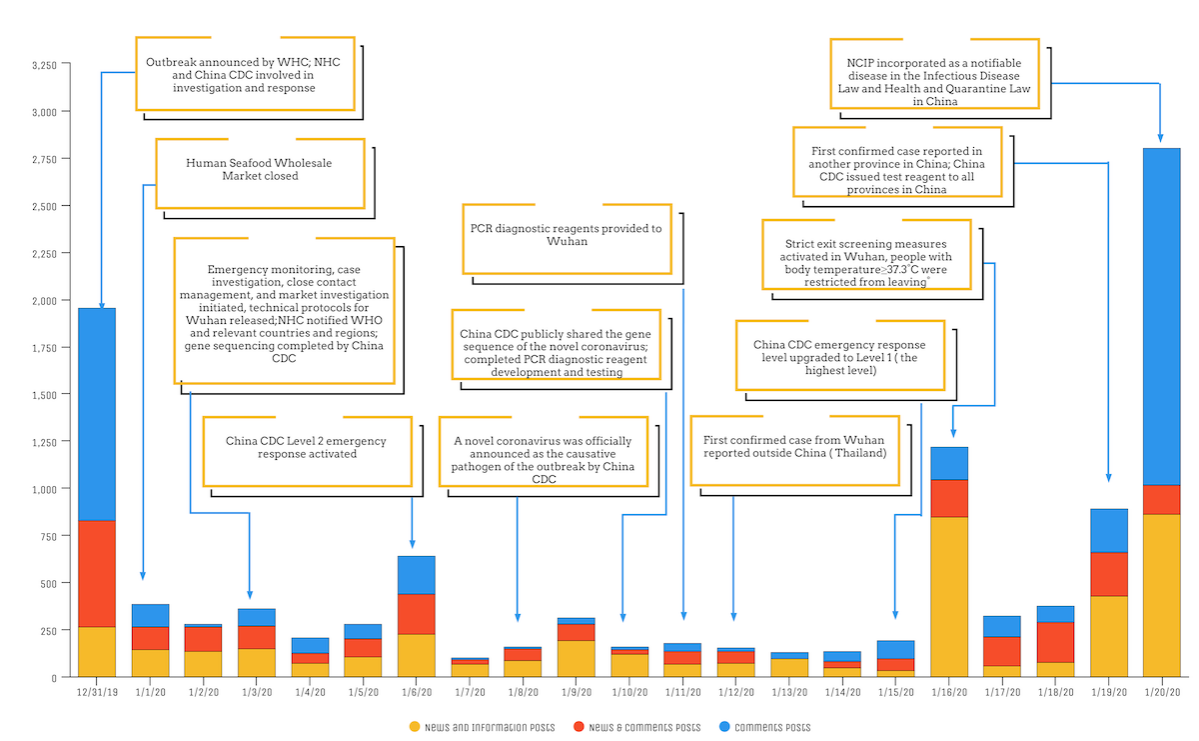
The number of Weibo posts related to the COVID-19 outbreak by information source category and with a timeline of events (December 31, 2019, to January 20, 2020). CDC: Center for Disease Control and Prevention; NCIP: novel coronavirus-infected pneumonia; NHC: National Health Commission; PCR: polymerase chain reaction; WHC: Wuhan Health Commission; WHO: World Health Organization.

The largest volume of daily posts (n=2804) was on the last day of our study period (January 20, 2020), and the lowest volume (n=101) was on January 7, 2020, with the average posts per day at 484 (SD 684.12, median 280). Among the 10,159 relevant posts, 4155 (40.90%) were government information and news and media source only posts, 3330 (32.78%) were user-generated comment posts, and 2674 (26.32%) contained both government and media information, and user-generated content concurrently (see Figure 1).

Results from our content analysis were broken up into findings describing our three parent classification areas: news and knowledge, public sentiment, and public reaction. News and knowledge were generally defined as posts containing COVID-19 outbreak information that originated from different information sources. In contrast, public sentiment was defined as the general attitude and sentiment of users in reaction to news and official government information sources about COVID-19. Finally, public reactions reflected actions or behaviors taken and self-reported by users in response to exposure to COVID-19 information. We noted that the three parent classifications areas often occurred concurrently in a single post, leading to a single Weibo post being classified for multiple parent classifications and subtopics.

### News and Knowledge

We retrieved and coded 64 unique news and knowledge posts, and the related subtopics comprised of a total of 6829 posts (67.22% of all 10,159 posts in this study; see Textbox 2 for post examples and Textbox 3 for representative subtopics identified). We then categorized all of these news and knowledge topics into four main thematic areas: (1) news and knowledge related to the causative agent of the disease (including the source of a novel coronavirus, the process to identify the causative agent, transmission pathway, and the naming of a novel virus), (2) epidemiological characteristics of the outbreak (including the number of confirmed cases, patients under intensive care, mortality, suspected cases, cases detected in and out of Wuhan, and cases reported in other countries), (3) official or personal recommended protective measures (including recommendations from the government and personal decisions on seeking protection from exposure to the virus), and (4) information on the government’s actions taken to address the COVID-19 outbreak.

Out of the 6829 posts containing news and information, we identified 18 subtopics from 2997 (43.89%) posts for categories under the first thematic area (causative agent and disease) based on our inductive coding scheme. These posts occurred over 16 days of the total 21-day study period. In the second category (epidemiological characteristics of the outbreak), we identified 24 topics from 3726 (54.56%) posts identified over a 20-day period. In the third thematic area (effective protective measures), we identified 3 topics from 375 (5.49%) posts, which occurred over only 5 days in our data collection period. Finally, the last thematic area (information on the government’s actions toward the COVID-19 outbreak) covered 19 topics from 4155 (37.08%) posts and occurred over a total of 14 days.

**Example language from posts for each study thematic area (with English translation).**

**News related to the causative agent**

- “截至7日21时，专家组认为，本次不明原因的病毒性肺炎病例的病原体初步判定为新型冠状病毒。四种冠状病毒在人群中较为常见，致病性较低，一般仅引起类似普通感冒的轻微呼吸道症状。另外两种冠状病毒——严重急性呼吸综合征冠状病毒和中东呼吸综合征冠状病毒，也就是我们简称的SARS冠状病毒和MERS冠状病毒，可引起严重的呼吸系统疾病。引起此次疫情的新型冠状病毒不同于已发现的人类冠状病毒，对该病毒的深入了解需要进一步科学研究。”
- English translation: “As of 21:00 on the 7th, the expert group believes that the pathogen of this unexplained viral pneumonia case is initially determined to be a new coronavirus. There are four coronaviruses are more common in the population and are less pathogenic, generally causing only mild respiratory symptoms similar to the common cold. The other two coronaviruses—Severe Acute Respiratory Syndrome Coronavirus and Middle East Respiratory Syndrome Coronavirus, which we refer to as SARS coronavirus and MERS coronavirus, can cause severe respiratory diseases. The new coronavirus that caused the outbreak is different from the human coronavirus that has been discovered, and further understanding of the virus requires further scientific research.”

**The number of confirmed cases, patients under intensive care, mortality, suspected cases, and cases detected in and out of Wuhan**

- “国家、省市专家组对收入医院观察、治疗的患者临床表现、流行病学史、实验室检测结果等进行综合研判，初步诊断有新型冠状病毒感染的肺炎病例41例，其中已出院2例、重症7例、死亡1例，其余患者病情稳定。所有密切接触者739人，其中医务人员419人，均已接受医学观察，没有发现相关病例。”
- English translation: “The national, provincial and municipal expert groups conducted comprehensive research and judgment on the clinical manifestations, epidemiological history, laboratory test results, etc. of patients admitted to the hospital for observation and treatment, and initially diagnosed 41 cases of pneumonia with new coronavirus infection, including 2 discharged, 7 severe cases and 1 death. The remaining patients were in stable condition. All 739 close contacts, including 419 medical personnel, have received medical observations and no related cases have been found.”

**Personal recommended protective measures**

- “希望大家重视，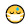 重在预防，戴口罩戴口罩戴口罩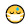 ·早晚各量一次体温 · 出门戴口罩，勤洗手 · 咳嗽或打喷嚏时捂住口鼻 · 将肉蛋彻底做熟 · 避免与呼吸道患者密切接触 · 避免近距离接触野生动物或活牲畜 · 不要随地吐痰 ·尽量避免人流量密集场所”
- English translation: “I hope everyone pays attention to it, 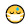 Focus on prevention, wear a mask, wear a mask and wear a mask 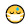 · Take your temperature in the morning and evening, wear a mask when going out, wash your hands frequently, cover your nose and mouth when coughing or sneezing, cook the meat thoroughly, avoid the respiratory tract Close contact with patients · Avoid close contact with wild animals or live animals · Don't spit anywhere · Try to avoid crowded places”

**Government’s actions taken to address the COVID-19 outbreak**

- “9日，湖北省卫健委称，武汉机场、铁路、公路等多地开始对人群进行体温检测。武汉12306热线表示，旅客进站会有热敏仪器对体温进行检测。”
- English translation: “On the 9th, the Hubei Provincial Health Municipal Commission said that Wuhan Airport, railways, highways and other places began to carry out temperature tests on the crowd. Wuhan 12306 hotline said that passengers will have thermal instruments to detect body temperature when entering the station.”

**Representative topics and subtopics for the news and knowledge category.**

**Causative agent and disease (December 31, 2019, to January 20, 2020)**

- Knowledge about “pneumonia of unclear cause,” “SARS” (severe acute respiratory syndrome), “MERS” (Middle East respiratory syndrome), and coronavirus
- Discussion about human-to-human transmission
- Ruled out seasonal influenza, avian influenza, SARS, MERS, and other common respiratory pathogens from potential causative agents
- Symptoms showed in confirmed cases
- Causative agent of the disease could originate from wildlife
- Causative agent preliminary identification of a novel coronavirus and named as 2019-nCoV
- Family cluster spread

**Epidemiological characteristics of outbreak (December 31, 2020, to January 20, 2020)**

- Suspected and confirmed cases in and outside of Wuhan
- Discussion about if there is any health worker infection cases reported
- Pneumonia of unknown etiology detected in Wuhan City
- Health status of patients
- Number of people under public health supervision because of contact with confirmed cases
- Death cases reported in Wuhan
- Patients reported having contact with Wuhan Seafood Wholesale Market
- Total number of confirmed cases, total number under intensive care, number of people under public health quarantine, and total number of deaths announced by Wuhan Health Commission

**Official or personal recommended effective protective measures (January 4-11, 2020**)

- Recommend people to wear masks, avoid crowds, and wash hands
- Traditional Chinese medicine to prevent infection
- Recommend people do not travel to Wuhan

**Government’s reaction to COVID-19 outbreak (December 31, 2019, to January 20, 2020)**

- Wuhan Central Hospital announced that SARS confirmed cases in the hospital was not true
- Official announcement of updated information related to “pneumonia of unclear cause”
- Mask shortage happened in some areas
- Investigation in Wuhan Seafood Wholesale Market
- World Health Organization advises against the application of any travel or trade restrictions on China
- China and other country governments’ reaction and response toward COVID-19

After identifying all subtopics, we then filtered based on the first date posted and identified four distinct timelines, one for each thematic area. In the first thematic area related to the causative agent and disease source, we observed an evolving timeline where the public gained understanding of what disease they were facing. “Pneumonia of unknown cause” was the first term people used to describe the outbreak, but this changed as the National Health Commission gradually ruled out seasonal influenza, avian influenza, and other common respiratory pathogens including Middle East respiratory syndrome (MERS) and SARS [28]. Finally, on January 9, 2020, the causative agent was identified as a novel coronavirus, though it was not until January 14, 2020, that the World Health Organization (WHO) announced the official name as 2019-nCoV (novel coronavirus) [29]. This timeline represents a period of initial uncertainty about the disease and its novelty.

Realization that COVID-19 could sustain human-to-human transmission was another critical event that emerged in Weibo posts. In the second timeline related to epidemiological characteristics, in the first week of our study, people who had close contact with the Wuhan Seafood Wholesale Market reported receiving a diagnosis of pneumonia of unknown origin, and the number of confirmed cases reported started increasing, as did the number of reported deaths. As early as day 5, suspected cases began being reported in countries outside of China (in Singapore and South Korea). On January 13, 2020, the first case outside of China was officially reported in Thailand [30]. Domestic spread was also reported, as Beijing and Guangdong started reporting confirmed cases toward the end of this timeline. Hence, this period represents early recognition of the emergence of a potential pandemic.

In the third timeline related to prevention (also coinciding with greater awareness of the causative agent and regional spread in timelines one and two), on January 5, 2020, the first news information that recommended implementing personal protective measures was detected. Recommendations included posts about wearing masks, avoiding crowds, and washing hands with soap and water or using alcohol-based hand gels. A few posts also appeared in the unofficial media claiming that Chinese traditional medicines could provide effective protection against COVID-19 infection. This period coincides with emerging information about COVID-19 and its spread, and can generally be viewed as the initiation of basic public health measures in an effort to contain what was now a known novel viral disease.

Starting from January 11, 2020, the government started to recommend avoiding travel to Wuhan. In this final timeline, posts showed a series of progressively restrictive policies issued from the government. During this time, the Chinese government also sent experts to Wuhan to help the local authorities investigate the outbreak and to prepare work related to outbreak control. This contrasted with government posts at the beginning of the outbreak that focused on information such as reports of the causative agents, announced closure of the Wuhan Seafood Wholesale Market (which at the time was considered the origin of the virus), and encouraging people to stop spreading unconfirmed information about the outbreak. Hence, this last timeline represents growing recognition of the seriousness of the outbreak and need for government intervention and more stringent control measures.

Finally, among all posts reviewed, we also observed some information that can be categorized as misinformation, particularly in the context of what is known about the disease now. For example, in user discussions about the causative agent, there were posts that believed that the SARS coronavirus was the causative agent. Another misinformation category was detected in user personal recommendations regarding effective disease preventative measures. For example, we detected posts suggesting that using BanLanGen (a traditional medicine) could prevent COVID-19 infection, even though there was no scientific basis at the time for this claim and no new evidence has emerged regarding its use to prevent or treat COVID-19 (also discussed in the *Public Reaction* section). Higher volumes of COVID-19 misinformation were not detected, likely due to these Weibo conversations occurring during the early stages of the pandemic, when there was insufficient basic information about the disease to generate misinformation or conspiracy-related topics. Content moderation and censorship may have also influenced the possible detection of misinformation on Weibo [31].

### Public Sentiment

Of the total 10,159 posts, we identified 7083 (69.72%) Weibo posts that included user public sentiment (which includes user comments and reactions to news and official reports). For this content, we focused on identification of specific user sentiment and detected 5 general classifications. Most of the user sentiment falls under the general category of anxiety about COVID-19, including expressions of uncertainty; being scared, worried, and nervous; and cautious sentiment. More strongly detected negative sentiment included expressions of anger. In contrast, there was also some general positive sentiment including those who expressed calm and optimistic attitudes in response to the outbreak.

**Figure 2:**
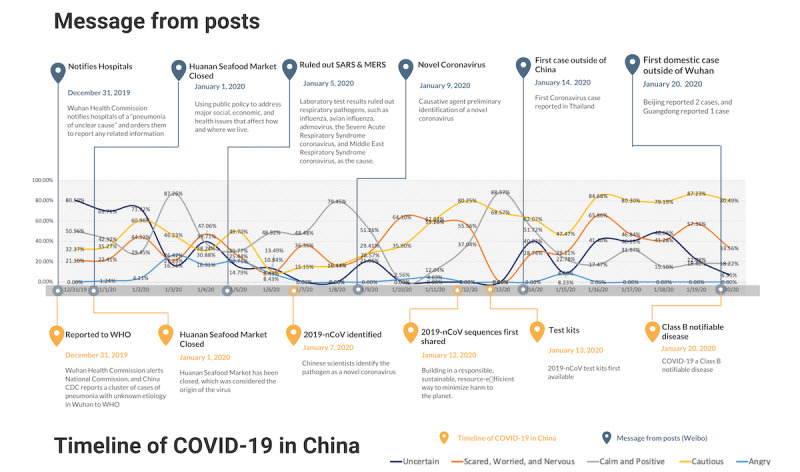
Percentage and classification of public sentiment posts and timeline of events. CDC: Center for Disease Control and Prevention; MERS: Middle East respiratory syndrome; SARS: severe acute respiratory syndrome; WHO: World Health Organization; 2019-nCoV: novel coronavirus.

In the general category of anxiety, uncertainty was the dominant sentiment detected. Expressions of uncertainty included questioning whether an infectious disease outbreak was underway, assessing the cause of pneumonia, speculation about the route of transmission, opinions about effectiveness of protective measures, and questioning the source of the outbreak. Of the 7083 posts identified as public sentiment, there were 2519 (35.56%) posts exhibiting uncertain sentiment throughout this 21-day period. The largest volume of these posts (n=1358) occurred in the beginning of the study period from January 3 to 13, 2020, when information about COVID-19 was still scarce and developing (see Figure 2).

Of the 7083 posts identified as public sentiment, the scared, worried, and nervous categories of the general anxiety sentiment included 2337 (32.99%) posts that included user conversations expressing concern and worry about the disease, other people not wearing masks in public areas, nervousness about one’s own health conditions, and worrying about family members. This sentiment was persistent from the first day until the last day of our study period but also varied in frequency, with the highest percentage of this sentiment detected on the last day of our study period, and the lowest frequency on January 13, 2020, when no posts for this sentiment were detected. This may be explained by the facts available at the time, including that on or around January 13, the public knew SARS had been ruled out as a potential causative agent; news about eight patients being discharged from a Wuhan hospital was released; and at the time, there was no evidence of human-to-human transmission.

Of the 7083 posts identified as public sentiment, caution was another anxiety-related sentiment that was detected with a total of 4073 (57.50%) posts and that steadily increased during the 21-day study period examined. In this category, users were cautious and reported that they would need to be prepared if human-to-human transmission of COVID-19 was confirmed. Additionally, after Chinese scientists identified the pathogen as a novel coronavirus, the percentage of posts with cautious attitude began to grow. Generally, as more knowledge about the COVID-19 outbreak and its disease etiology became available, more users expressed cautious sentiment.

In the stronger negative sentiment category of the 7083 posts identified as public sentiment, those expressing anger were detected in only 177 (2.5%) posts, spanning from days 8-21 of the study period. Among these posts, 16 complained about the slow reaction from the Chinese government in response to the outbreak, and 161 posts expressed anger about other people not wearing masks in public areas. Hence, overall criticism of the Chinese government’s COVID-19 response appeared to be minimal, while the vast majority of anger-related posts concerned other members of the public who were perceived as creating higher risk of transmission.

In contrast to the general anxiety and anger-related sentiment of users from the 7083 posts identified as public sentiment, we also detected 177 (2.50%) posts reflecting positive sentiment about the outbreak. These users expressed calm and optimism about outbreak conditions, including users who were generally not worried about the seriousness of the outbreak, believed the government and current medical technology was sufficient to control the outbreak, and reported being satisfied with the level of transparency of the Chinese government and its outbreak response. Overall, positive sentiment posts were much lower in volume compared to another user sentiment detected.

Among these sentiment categories, statistically significant stationarity over time was observed for percentage of posts in the “calm and optimistic” category (*P*<.0001) and the “angry” category (*P*<.0001; see Table 1). Percentage of posts in the “cautious” category exhibited significant linear and exponential relationships, with exponential fit appearing to be slightly better than linear fit (*R*^2^: 0.55 vs 0.50). Percentage of posts in the “uncertain” category exhibited a significant exponential trend (*R*^2^=0.32) but not a linear trend. Though nonstationary, percentage of posts in the “scared, worried, and nervous” category exhibited neither linear nor exponential significant trends. These results indicate that people in Wuhan became dramatically more cautious and consistently less uncertain during this 21-day period.

**Table 1:** Results of eight sets of regression models, corresponding to each of the observed attitudes and reactions from Weibo posts, with each set containing a linear model and an exponential model.

Dependent variable	Independent variable	Posts, n	Linear				Exponential^a^		
			β	*P* value	*R^2^*	*b*		*P* value	*R^2^*
Uncertain (%)	Date	21	–0.0145	.12	0.12	–0.2260		*.004* ^b^	0.32
Scared (%)	Date	21	0.0104	.11	0.12	0.0286		.13	0.59
Cautious (%)	Date	21	0.2819	*<.001*	0.50	0.0596		*<.001*	0.54
Mask (%)	Date	21	0.0168	.07	0.16	0.0372		*.04*	0.18
Cancel (%)	Date	21	–0.0001	.98	<0.01	–0.0034		.95	<0.01
Self-treatment (%)	Date	21	–0.0003	.20	0.08	–0.1135		.42	<0.01
Normal (%)	Date	21	–1.2999	.99	<0.01	–0.0006		>.99	<0.01
Evacuate (%)	Date	21	–0.0006	.44	0.03	–0.1647		.09	0.12

^a^The exponential coefficient and *P* values were provided for the b coefficient in the *Y=a+b^x^* equation, and exponential *R*^2^ values were computed as Cox and Snell *R*^2^.

^b^Italics represent statistically significant *P* values.

### Public Reaction

**Figure 3:**
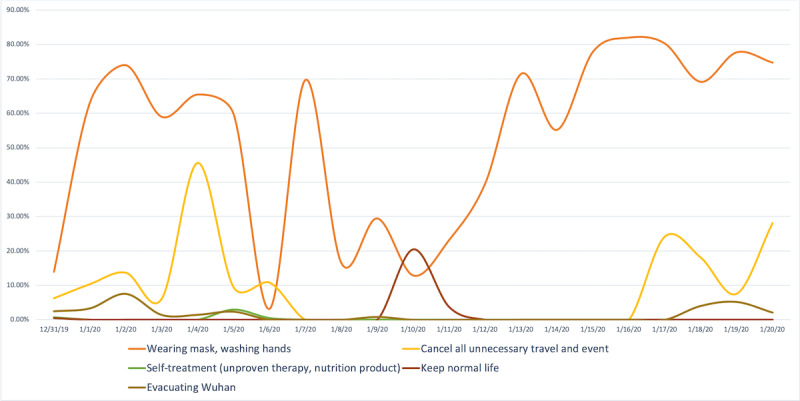
Percentage of posts classified as public reaction and their categories.

In the last category of post characteristics, of the total 10,159 posts, we detected 4863 (47.87%) posts that self-reported user reaction to COVID-19–related information, which also led to identification of five different types of resultant behaviors reported by users (see Figure 3). Reactions included self-reporting protective behavioral factors including wearing masks; washing hands more frequently; and cancelling all unnecessary travel, gatherings, and events. In contrast, some self-reported behavior we detected could be categorized as elevating health risk, including self-treatment with unproven therapy and nutritional products, maintaining preoutbreak lifestyle and habits, and self-evacuation from Wuhan. We noted that some of these reaction themes also occurred concurrently.

For example, a total of 3689 posts (75.86% of all public reaction posts) included users reporting their choice to start wearing masks. This behavior also coincided with public announcements, with an increase of approximately 50% in the number of mask-related posts observed 2 days after such recommendations were made. However, on January 6, 2020, the percentage of mask-related posts dropped to 3.13%. Based on our timeline of events, we observed that on January 5, 2020, the Chinese health commission ruled out SARS and MERS as the causative agent, which could have led to a sense of false security and lower mask use among the public. On January 7, when the causative agent was identified as a novel coronavirus, the percentage of mask posts then rose to 69.70% and continued to fluctuate based on other news events.

The second largest volume of public reaction posts (n=987, 20.30% of all reaction posts) was related to canceling unnecessary travel and events, followed by evacuating Wuhan (n=148, 3.04%), maintaining a normal lifestyle (n=20, 0.41%), and beginning self-treatment (n=19, 0.39%; see Textbox 4 for examples). These posts evidenced that users not only were exposed to and changed their knowledge, attitudes, and perceptions about the outbreak but also acted upon changing information through different types of behaviors and actions. Some of these reactions could have directly impacted outbreak control measures (eg, traveling when not recommended prior to an official quarantine, attending public gatherings, and engaging in self-care).

No public reaction category exhibited statistically significant stationarity (see Table 1). Percentage of posts in the “wearing mask, washing hands” category exhibited a significant exponential trend over time (*R*^2^=0.19) but not a linear trend. Other categories did not exhibit significant linear or exponential trends. Over this 21-day period, the Wuhan population appears to have gravitated toward wearing masks and washing hands but not toward other preventative behaviors.

**Example language from posts for each type of behaviors.**

**Self-reporting protective factors**

- Wearing masks and washing hands
  “除了不传谣不信谣, 自己平常多注意少去人多密集的地方, 有症状及时就医 老百姓做的还能有啥, 难道真要等到武汉封城才开始注意吗? 在网上乱猜想还不如多喝热水, 多带口罩, 多睡觉!”
  English translation: “As a normal citizen, there is nothing we can do, except not spreading rumors, not going to crowed, and going to hospital when symptoms shows. Shouldn’t have to wait until the lock down of Wuhan, people will start paying attention? It works better to drink more hot water, wear mask and sleep more rather than making assumption online.”
- Cancelling all unnecessary travel, gatherings, and events
  “因为武汉新型冠状病毒肺炎，不得不取消回武汉的行程. 很久很久没见父母亲人了. 突然好难过，退机票的时候没忍住哭了”
  English translation: “Because of Wuhan's new coronavirus pneumonia, I had to cancel my trip back to Wuhan. I haven't seen my parents and relatives for a long time. Suddenly feeling so sad and couldn't help crying when refunding the ticket.”

**Behavior that could elevate health risk**

- Self-treatment with unproven therapy and nutritional products
  “赶紧掏出板蓝根，管他有没有用”
  English translation: “Find out Banlangen (a traditional Chinese herbal medicine. It has been used for the prevention and treatment of virus-related respiratory diseases such as influenza virus infection [32]. whether it is useful or not.”
- Maintaining preoutbreak lifestyle/habits
  “病毒也不能阻止我，戴上口罩去跨年”
  English translation: “The virus can't stop me, put on a mask to go to the New Year celebration.”
- Self-evacuation from Wuhan
“火灾+地震+不明原因肺炎, 还是逃离武汉吧”
  English translation: “Fire + earthquake + unknown caused pneumonia, should escape from Wuhan.”

## Discussion

### Principal Findings

Our study conducts an in-depth qualitative analysis of 10,159 COVID-19 Weibo posts from 8703 unique Sina users located at the initial epicenter of the outbreak, Wuhan City. In these posts, we observed that 67.22% (n=6829) of posts included news and knowledge posts, 69.72% (n=7083) included public sentiment topics, and 47.87% (n=4863) included public reaction and self-reported behavior. Though major thematic issues about the causative agent, epidemiological characteristics of the outbreak, and personal protective measures along with how the Chinese government responded to this novel virus were detected, these topics were not static and changed over time as new information about COVID-19 emerged and was communicated to the public. Initial uncertainty and changing knowledge, attitudes, and beliefs about COVID-19 also coincided with changes in users’ sentiment and self-reported behaviors that may have acted to mitigate or potentially worsen the spread of the disease.

This study is limited to 21 days of social media posts that occurred at the early stages of the COVID-19 outbreak in Wuhan, China to better understand key topics related to individuals’ awareness, concerns, and reactions to the crisis. Overall, the volume of posts varied throughout the study period, with the largest volume of posts originating from government or news and media only sources. We also observed a high volume of uncertainty sentiment about COVID-19 from Weibo users in Wuhan, though these expressions of uncertainty varied and gradually cleared with time. We observed different timelines of events that related to specific news and knowledge categories beginning with uncertainty about COVID-19; growing concern of a potential global pandemic; and finally, initial government and public actions to contain the spread of the disease, indicative of an early outbreak public reaction and response.

Specifically, one of the most consequential announcements marked the beginning of the pandemic timeline and occurred on December 31, 2019, when the Wuhan Health Municipal Commission announced the emergence of a pneumonia and respiratory illness of unknown origin, followed by a similar announcement by the WHO 6 days later [33,34]. Following this announcement, several key early outbreak events occurred, including the reporting of the Huanan Seafood Market as the suspected origin of the outbreak, confirmation that the virus was a novel coronavirus, establishment by the Chinese government of public health and sanitation guidelines, and the announcement of human-to-human transmissibility of COVID-19 [35]. All these events appeared to influence the news and information disseminated on Weibo and impacted the public’s sentiment and reaction to COVID-19. Our study period ended on another important early outbreak event: the implementation of the quarantine of Wuhan City on January 23, 2020, where we observed a rapid increase in social media conversations but that were not analyzed for this study.

These findings also indicate that the relationship between exposure to news and information also fits well with the agenda-setting theory, which describes how the media stimulates awareness, shapes and filters reality, and sets priorities of the public for salient issues including for public health concerns [36]. In this case, the exposure to changing news and information on Weibo and the ability of these social media users to directly communicate their opinions and behaviors evidenced the complex interaction between the government, media sources, and the public during an early outbreak period. This interaction is supported by past studies that have also found an association with an increase in the volume of social media posts about an outbreak and major news events in past health emergencies [14,37]. Our findings provide further clues about how the public responds to disease outbreak communication and specific events as they unfold, which can increase anxiety and even misplaced optimism about the personal and population health risk of a novel disease. Future work should focus on further adapting the agenda-setting theory to health promotion efforts targeted for outbreak response and that is contextualized for local communities and social media platforms [36].

Importantly, this study provides early insight into COVID-19, the most consequential disease outbreak in the last century, which occurred at a time when global public engagement on social media platforms such as Weibo are at an all-time high. Analyzing social media data can provide valuable insights into a communities’ knowledge, concerns, and fears, which can influence individual and population-level behavior—important factors that can have a direct impact on the success or failure of public health interventions aimed at containing the spread of a disease. These findings can also aid in developing communication tools and health promotion activities to help the public better understand transmission risks, correct confusion or misinformation, and educate on social and behavioral risks that may exacerbate spread.

Though our study was limited to Wuhan City and the early stages of the outbreak, these infoveillance insights are salient even now. This includes using this information to better prepare for re-emergence or new waves of COVID-19 in different communities; ensuring appropriate health messaging on new COVID-19 developments such as vaccine and therapeutic deployment; and communicating to the public about ongoing social distancing, quarantining, masking, and reopening recommendations. Hence, the importance of the “infoveillance” field for outbreak detection and monitoring has arguably never been more important, with this study representing one piece of this growing field that can generate closer to real-time public health intelligence from digital data sources. It is our hope that these results can help inform governments and public health stakeholders on strategies to improve outbreak communication for COVID-19 and into the future, in an era where digital platforms are now a dominant source of information and interaction.

### Limitations

This study has certain limitations. Our data collection was limited to a single Chinese social media platform and to a specific geographic area. Hence, the findings are in no way generalizable to all COVID-19 social media conversations occurring among Chinese users. Our data collection focused on the early stages of the COVID-19 outbreak. However, during periods of this time frame, the causative agent had not been confirmed, and there was no official name for the disease. Due to this early inconsistency in disease terminology, Weibo users may have used other keywords to describe COVID-19–related conversations or topics that were not collected by this study. Finally, due to possible censorship of social media posts, some Weibo posts may have been deleted before data collection, and these conversations or public sentiment may not have been captured.

## Abbreviations

MERS: Middle East respiratory syndrome
SARS: severe acute respiratory syndrome
WHO: World Health Organization
2019-nCoV: novel coronavirus
